# Brand new norms for a good old test: Northern Italy normative study of MiniMental State Examination

**DOI:** 10.1007/s10072-021-05845-4

**Published:** 2022-01-06

**Authors:** Giuseppe Foderaro, Valeria Isella, Andrea Mazzone, Elena Biglia, Marco Di Gangi, Fabrizio Pasotti, Flavia Sansotera, Monica Grobberio, Vanessa Raimondi, Cristina Mapelli, Francesca Ferri, Valentina Impagnatiello, Carlo Ferrarese, Ildebrando Marco Appollonio

**Affiliations:** 1 EOC Ente Ospedaliero Cantonale, Lugano Regional Hospital, Lugano, Canton Ticino, Switzerland; 2 Neurology Department, San Gerardo Hospital, Monza, School of Medicine, University of Milano-Bicocca, Milan, Italy; 3 Milan Center for Neurosciences (NeuroMI), Milan, Italy; 4 Redaelli Geriatric Institute, Milan, Italy; 5 Neurocenter of Southern Switzerland, Lugano Regional Hospital, Lugano, Canton Ticino, Switzerland; 6 Psychology, Psychotherapy and Neuropsychology Center, Canton Ticino, Minusio Switzerland; 7 Department of Brain and Behavioural Sciences, University of Pavia, Pavia, Italy; 8 Clinical Neuropsychology Lab, Neurology Department and Clinical Psychology Unit, ASST Lariana, Como, Italy; 9 Psychology Service and Neurology Unit, ASST Crema, Crema, Italy

**Keywords:** MiniMental State Examination, MMSE, Normative study, Cognitive screening, Neuropsychological assessment

## Abstract

**Aim:**

Mini-Mental State Examination (MMSE) is one of the most used tests for the screening of global cognition in patients with neurological and medical disorders. Norms for the Italian version of the test were published in the 90 s; more recent norms were published in 2020 for Southern Italy only. In the present study, we computed novel adjustment coefficients, equivalent scores and cut-off value for Northern Italy (Lombardia and Veneto) and Italian speaking Switzerland.

**Methods:**

We recruited 361 healthy young and old (range: 20–95 years) individuals of both sexes (men: 156, women: 205) and from different educational levels (range: 4–22 years). Neuropsychiatric disorders and severe medical conditions were excluded with a questionnaire and cognitive deficits and were ruled out with standardized neuropsychological tests assessing the main cognitive domains. We used a slightly modified version of MMSE: the word ‘fiore’ was replaced with ‘pane’ in verbal recalls to reduce the common interference error ‘casa, *cane*, gatto’. The effect of socio-demographic features on performance at MMSE was assessed via multiple linear regression, with test raw score as dependent variable and sex, logarithm of 101—age and square root of schooling as predictors.

**Results:**

Mean raw MMSE score was 28.8 ± 1.7 (range: 23–30). Multiple linear regression showed a significant effect of all socio-demographic variables and reported a value of R^2^ = 0.26. The new cut off was ≥ 26 /30.

**Conclusion:**

We provide here updated norms for a putatively more accurate version of Italian MMSE, produced in a Northern population but potentially valid all over Italy.

## Introduction

In 1975, Marshal and Susan Folstein, from the New York Hospital-Cornell Medical Center in NY, USA, published a brief and simple tool for the assessment of ‘mental state’ in patients with dementia or psychiatric disorders, the Mini-Mental State Examination (MMSE) [10]. The scale includes the following subtests: orientation in time (year, season, date, day of the week, month) and space (state, county, town, hospital, floor); immediate and delayed repetition of three words (specified in a later paper [30]: ‘apple, penny, table’); attention and mental calculation (serial subtraction of 7 s from 100 and/or backward spelling of the word ‘world’); confrontation naming (of a pencil and a wrist watch upon visual presentation); sentence repetition (‘No ifs, ands or buts’); comprehension and execution of oral (‘Take a paper in your right hand, fold it in half and put it on the floor’) and written (‘Close your eyes’) commands; writing of a sentence containing a subject and a verb; copy of two intersecting pentagons. One point is attributed to each correct response, for a total score of 30. The cutoff for normality was set at 24/30 based on the lowest score obtained by a group of healthy seniors (Folstein 1975). Since then, the popularity of MMSE has grown to the point that the term ‘MMSE’ now yields nearly 20,000 Pubmed records and has gone from 14 citations in 1975 to more than 1400 in 40 years, in 2015. The test has in fact been used and is still widely used, for the screening of global cognition in a great variety of medical and neurological populations (e.g. patients with stroke [29], Multiple sclerosis [25], hepatic encephalopathy [16] or renal failure [8], just to mention a few), with dementia being its main and most successful area of application [7, 17].

The popularity of the test is justified by its ability to provide a simple, rapid (it takes 5 to 10 min to administer) and generally reliable evaluation of cognitive functioning. Even if limitations have emerged that should be taken into account by clinicians, e.g. the lack of items assessing executive abilities or the poor sensitivity to mild cognitive impairment (MCI) [12, 18], and have driven the development of alternative, promising tools like the Montreal Cognitive Assessment (MOCA) [21], the MMSE is still the most widespread cognitive screening test. Considering its main field of application, dementia, a recent overview of the neuropsychological tests used in memory clinics conducted by the Italian National Institute of Health in 501 centres established that, together with the Clock drawing, MMSE is the tool used most frequently for the screening of dementia [9]. In fact, it represents the official reference tool for prescription and monitoring of anticholinesterase inhibitors in Alzheimer’s disease indicated by Italian health authorities [3].

The MMSE has been translated and validated in a number of countries [1, 2, 14, 24]. Its first Italian translation appeared in 1993, in a paper reporting findings about the relationship between global cognition as measured by the test and lifetime occupational activity in an elderly sample [11]. In this version of the test, county was replaced by region, the three bi-syllabic high frequency words ‘casa, pane, gatto’ (house, bread, cat) were used for immediate and delayed recalls, the word ‘carne’ (meat), whose phonemic complexity is similar to the original item ‘world’, was used for backward spelling and the tongue twister ‘Sopra la panca la capra campa’ (above the bench the goat lives) was employed in the sentence repetition subtest. Although other variants of the test have been proposed later, only this version has undergone a normative study. More precisely, three normative sets have been produced for the Italian MMSE, two were published in the 90 s and still largely in use [19, 20] and one was published in 2020[4, 5].^1^ All three studies have applied the same statistical design, i.e. computing adjustment coefficients and a cut point using multiple linear regression analysis (Carpinelli Mazzi et al. also provided equivalent scores), but differ in several population characteristics (Table 1). The study by Measso et al. was performed in a sample of approximately 900 individuals aged 20 to 79 years and selected from population registers of seven communities across Italy and San Marino and screened with a medical interview for excluding dementia, mental insufficiency, psychiatric disorders and other conditions potentially affecting the cognition. The study by Magni et al. included approximately 1000 participants from Lombardia, in the North of Italy, also sampled from local registers, but the age coverage was limited to older decades (65–89 years). The only exclusion criterion was, oddly, MMSE itself (namely a raw score < 21). The study by Carpinelli Mazzi et al. also used MMSE as an exclusion criterion, precisely a score = 0 on the delayed recall of the three words, in addition to a health questionnaire, and included slightly more than 300 individuals aged 50 years and over through general practitioners or memory clinics in Campania, in the South of Italy. Age and education, not sex, were found to affect the performance on MMSE in all three studies, but regression models yielded different correction values, and also different cut off values which, for some socio-demographic slots, are only partly counterweighed by the differences in cut points. Hence, application of different norms may lead to a discrepant classification of a patient’s performance, especially for borderline scores. As an example, a raw MMSE score of 23 for a 70-year-old individual with 8 years of schooling would be above the cutoff for normality according to Measso’s norms (adjusted score: 24.20, cut off ≥ 23.8) but below according to norms by Magni (adjusted score: 21.40, cutoff ≥ 22.0) and Carpinelli Mazzi (adjusted score: 23.92, cutoff ≥ 24.9). A more extreme case is that of a 70-year-old individual with 3 years of schooling and a raw MMSE score of 20, who would be above the cutoff for normality according to Measso (adjusted score: 25.24, cut off ≥ 23.8), but below according to both Magni (adjusted score: 20.07, cutoff ≥ 22.0) and Carpinelli Mazzi (adjusted score: 21.54, cutoff ≥ 24.9).

**Table 1:** Characteristics of normative studies for Italian MMSE (a fourth study, by Grigoletto et al., was a re-analysis of Measso et al.’s data computing fifth percentile norms as step functions of age)

	Measso et al. [20]	Magni et al. [19]	Carpinelli Mazzi et al. (2020a, b
Geographic areas	Northern-Central-Southern Italy and San Marino	Lombardia	Campania
Enrolment source	Registry office	Registry office	General practitioner or memory clinic attendees
Selection methods	Medical history, Geriatric Depression Scale	MMSE (score ≥ 21)	Medical history, delayed recall of MMSE = 0/3
No. of participants (M/F)	906 (441/465)	1019 (350/769)	314 (161/153)
Age:			
Range	20–79 yrs	65–89 yrs	50–79 yrs
Mean ± SD	Not specified	75.4 yrs ± 5.4	63.4 yrs ± 9.0
Education:			
Range	0 +	0 +	3 +
Mean ± SD	8.4 yrs ± 0.1	5.2 yrs ± 2.5	11.5 ± 4.4
Age	Six decades	Five 5-year periods	Three decades
Education (years)	0–3, 4–5, 6–8, 9–13, ≥ 14 yrs	0–4, 5–7, 8–12, ≥ 13 yrs	3–5, 6–8, 9–13, ≥ 14 yrs
Mean MMSE score ± SD	27.7 ± 2.6	27.0 ± 2.4	27.8 ± 1.8
Statistical analysis	Multiple linear regression on age, sex and education	Multiple linear regression on age, sex and education	Multiple linear regression on age, sex and education
Cutoff	> 23.8	> 22.0	> 24.9

Periodic update of norms for neuropsychological tests is demanded first of all not only by longitudinal demographic changes in the reference population but also by the evolution of educational and cultural standards. In Italy, from 1981 to 2011, the proportion of citizens with a high school or a university degree has increased, respectively, from 11.5 to 30.2% and from 2.8 to 11.2% (https://www.istat.it/it/). In addition to a rise in formal schooling, the last three decades have also seen a strong growth in diffusion and access to culture, technology and digital communications, with a positive impact on global level of knowledge and cognitive skills of Italian general population [15, 27]. The primary aim of the current study was thus to update norms (adjustment coefficients, equivalent scores and cut off) for Italian MMSE in a sample of healthy young and old individuals from Northern Italy. In doing so, we chose ‘quality over quantity’, meaning that ruling out individuals with MCI (a concept developed after publication of all three Italian normative studies of MMSE) [23] was deemed more relevant than collecting a large but poorly screened sample size. Therefore, all participants underwent formal assessment of episodic memory, language production, executive functioning (lexical retrieval strategy and shifting) and visuo-constructional ability before being included in the study. The second objective was to compute extremely punctual correction coefficients that allowed a more tailored adjustment of individual raw scores on the test. We have therefore considered quite restricted socio-demographic ranges, in particular for tertiary school attainment levels, which have expanded greatly and have been substantially redesigned in the Italian educational system in the last few years. A third aim was to extrapolate norms also for the oldest-old, to keep up with progressive population ageing. A fourth and final objective was to develop a simple but well-defined administration and scoring protocol. Several versions of MMSE are in fact in use in Italy that differ in terms of administration procedure and content (e.g. asking for province instead of region, or using ‘mondo’ —world— for the spelling task and ‘tigre contro tigre’ —tiger against tiger— as tongue twister), sometimes have obscure administration and scoring instructions and, more importantly, do not correspond to the versions used in the normative studies and thus have never undergone formal standardization. In revising the test protocol, we also propose an amendment to the original version of the test: the word ‘pane’ has been replaced with ‘fiore’ in order to avoid the common error ‘casa, *cane* (dog), gatto’ induced by the phonemic assonance between ‘casa’ and ‘pane’ and the semantic relatedness between ‘gatto’ and ‘cane’.

## Subjects and methods

Study participants were recruited in three Northern Italian cities, Monza (San Gerardo Hospital), Como (Sant’Anna Hospital) and Bussolengo (Verona, Orlandi Hospital), and in Canton Ticino, a Swiss canton that shares international borders with Italy and has Italian as the sole official language. Individuals of both sexes, 20 years of age or older, and with Italian as their native language, could participate into the study. They completed a questionnaire about their medical and pharmacological history, also including a question about subjective cognitive complaints, and underwent the following standardized neuropsychological tests: clock drawing [26], logical memory (‘Anna Pesenti’) [22], category fluency (animals and fruits, 60 secs each) [31] and letter fluency (F-A-S, 60 secs each) [6]. The tests were administered on the same session of MMSE and of the health questionnaire. Exclusion criteria were a past or present history of neurological or psychiatric disorders, brain injury, mental insufficiency or learning disabilities, severe medical conditions, substance abuse, presence of significant motor or sensory deficits and an abnormal score on any one of the four co-administered neuropsychological tests.

Informed consent was obtained from all participants included in the study.

**Fig. 1:**
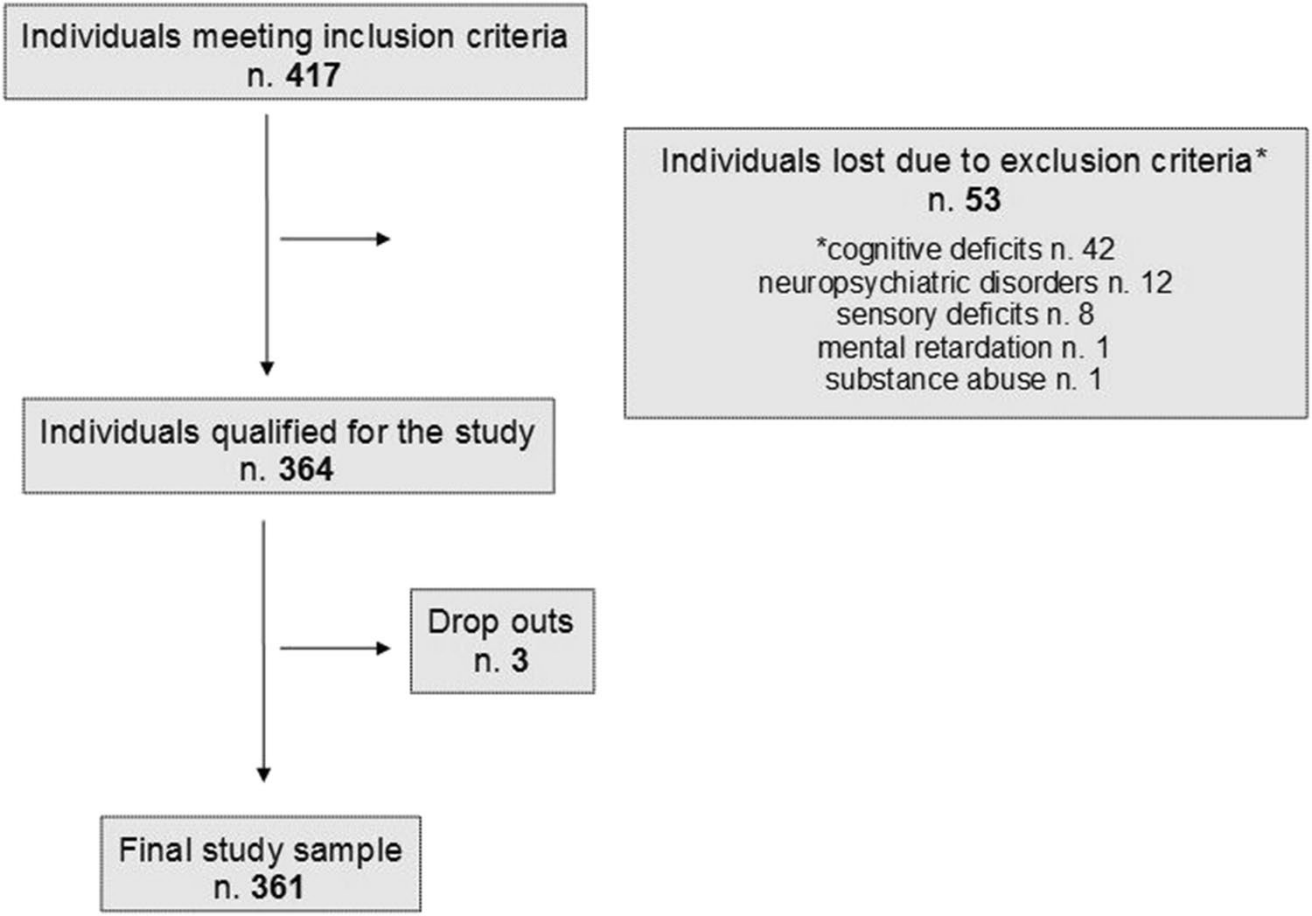
Algorithm of participant selection

Figure 1 summarizes the enrolment process. The final study population was composed by 361 individuals, after the exclusion of 53 subjects from an initial pool of 417 candidates. The main reason for the exclusion was the presence of cognitive deficits: 42/417 subjects (10.1%) showed one or more abnormal scores on the neuropsychological screening battery. The majority were enrolled from Canton Ticino (*n*. 180, 49.9%), followed by Monza (*n*. 115, 31.9%), Como (*n*. 49, 13.6%) and Bussolengo (*n*. 17, 4.7%).

Table 2 reports the socio-demographic characteristics and mean scores on the neuropsychological screening battery for the entire final study sample, and Table 3 the number of men and women for each age and education group. There was a slight prevalence of women (*n*. 205, 56.8%). Age range was 20–95 years and approximately half of the participants (*n*. 178, 49.3%) were aged 60 years or over, while educational range was 4–22 years and the majority of participants (*n*. 194, 53.7%) had less than 13 years of schooling (without major differences between men and women), but only two had less than 5 years of formal education. As per the increased schooling of the general population, only few subjects in the younger age ranges had a low educational level: none below the age of 45 had less than 8 years of schooling.

**Table 2:** Socio-demographic features and scores on the neuropsychological screening battery of the study population (scores are adjusted for sex, age or education, as appropriate)

	*Cut off*	Mean	Standard deviation	Median	Minimum	Maximum
Age	*-*	56.8	19.4	59	20	95
Education (years)	*-*	11.3	4.1	11	4	22
Clock drawing	> *4.9*	9.1	1.0	9.3	5.0	10
Logical memory	> *8.0*	14.1	3.1	14.2	8.0	24.3
Category fluency:						
Animals	> *9.6*	18.3	4.0	17.8	9.6	35.6
Fruits	> *7.5*	14.9	3.1	14.5	7.5	25.0
Letter fluency	> *17.8*	34.6	8.6	34.2	18.0	63.2

**Table 3:** Number of subjects for age *x* education slot (men/women = total)

Education (years of schooling)									
Age:	0–2	3–4	5	6–8	9–13	14–16	17–18	** > **19	Total
20–24	-	-	-	1/1	9/2	2/2	0/2	-	12/7 = 19
25–29	-	-	-	1/2	7/11	2/3	4/3	-	14/19 = 33
30–34	-	-	-	2/0	3/5	1/0	0/1	1/0	7/6 = 13
35–39	-	-	-	3/2	5/7	0/3	2/1	-	10/13 = 23
40–44	-	-	-	3/0	4/2	1/1	3/0	0/1	11/4 = 15
45–49	-	-	0/2	1/6	1/8	0/2	1/1	-	3/19 = 22
50–54	-	-	2/2	2/7	5/5	1/0	2/1	-	12/15 = 27
55–59	-	-	0/2	5/3	4/10	2/2	0/3	-	11/20 = 31
60–64	-	-	4/4	7/3	1/3	1/1	2/3	0/1	15/15 = 30
65–69	-	-	2/2	3/4	3/7	2/0	1/1	-	11/14 = 25
70–74	-	0/1	4/0	5/8	7/6	-	4/3	1/0	21/18 = 39
75–79	-	0/1	0/4	2/11	3/10	2/0	2/4	1/0	10/30 = 40
80–84	-	-	4/2	1/7	4/7	-	4/2	-	13/18 = 31
85–89	-	-	1/2	1/2	1/0	1/0	1/0	-	5/4 = 9
90–94	-	-	-	-	1/2	-	-	-	1/2 = 3
95–99	-	-	-	-	-	-	0/1	-	0/1 = 1
Total	-	0/2 = 2	17/20 = 37	37/56 = 93	58/85 = 143	15/14 = 29	26/26 = 52	3/2 = 5	156/205 = 361

### MMSE protocol

The integral version of MMSE used in the study is presented in the Appendix.

*Temporal orientation* was assessed asking the subject for (in administration order) date of the month, month, year, day of the week and season [score 0–5], and *spatial orientation* was assessed asking for (in order) place ‘hospital’, ‘clinic’ …), floor, town, nation and region (canton, for Swiss participants) [score 0–5].

The three words used for immediate [score 0–3] and delayed [score 0–3] *recall* were (in order) ‘casa’ (house), ‘fiore’ (flower) and ‘gatto’ (cat). At immediate recall; the score was attributed after the first repetition trial, but the stimuli were reproposed if the subject omitted one or more items until all three were repeated or up to a maximum of six trials overall. No warning was given about the delayed recall.

In the *attention and calculation* subtest, consecutive serial subtraction of sevens, from 100 to five subtractions, was performed first, and backward spelling of the word ‘carne’ (meat) was administered in case of one or more calculation errors, or if the subject proved completely unable to perform the calculation task. One point was attributed to each correct subtraction and to each letter produced in the correct position (e.g. *enrac* = 5 points, *ernac* = 3 points); the final score was the highest score achieved in either task [score 0–5].

Language subtests included the following: *naming* upon visual presentation of a pen (both Italian nouns for pen, ‘penna’ and ‘biro’, were accepted as correct, while the term ‘matita’ (pencil) was considered incorrect) and a wrist watch [score 0–2]; *repetition* of the sentence ‘Non c’è se ne ma che tenga’ (No ifs or buts) [score 0–1]; *comprehension* and execution of the spoken three-stage command ‘take this sheet of paper with your right hand, fold it in two and put it on the floor’ [score 0–3]; *reading* aloud and execution of written command ‘close your eyes’ [score 0–1]; *writing* of a sensible sentence containing a subject and a verb. In the latter subtest, an implicit subject was accepted as correct, and grammar errors were disregarded [score 0–1].

The model of intersecting pentagons used for the *copy* task is shown in the Appendix. Performance was considered correct when ten angles were present and two intersected in the shape of a four-sided diamond; minimal discontinuity in the lines at angles was accepted as correct [score 0–1].

### Statistical analysis

Statistical analysis was performed with SPSS version 27 (IBM Corp., Armonk, NY, USA).

Multiple linear regression was carried out to explore the relationship between MMSE score and age, sex and years of schooling. Various transformations of age and education (logarithmic, quadratic, inverted, subtraction) were considered to find the regression model accounting for the largest proportion of total variance. Each variable was evaluated separately and included in the final model if its significance was confirmed on simultaneous regression with the other significant single predictors. Values estimated by the regression model were then used for adjusting original MMSE scores for significant socio-demographic variables. For empty socio-demographic cells (e.g. education ≤ 2 years or age ≥ 100 years), adjustment values were *extrapolated* by regression analysis. A nonparametric method was applied to the adjusted scores ranked in increasing order to identify outer and inner one-sided tolerance limits for the lower 95% of the population, with a 95% confidence level. Finally, following a consolidated procedure [28], adjusted scores were transformed into equivalent scores whereby zero corresponds to a score below the 5% tolerance limit.

## Results

Average raw MMSE score for the entire study sample was 28.6 ± 1.7 (range: 23–30), with no statistically significant difference across enrolment centres (Canton Ticino: 28.5 ± 1.9, Monza: 28.8 ± 1.5, Como: 28.9 ± 1.6, Bussolengo: 28.7 ± 1.9; *p* = 0.224). See Table 4 for means and standard deviations of MMSE scores stratified by age and education.

**Table 4:** MMSE raw mean scores and standard deviations for each age x education slot (only values and slots with a minimum of two subjects are shown)

Education (years of schooling)					
Age:	5	6–8	9–13	14–16	17–18
20–24	-	29.5 ± 0.7	29.2 ± 0.9	29.8 ± 0.5	30.0 ± 0.0
25–29	-	29.7 ± 0.6	29.4 ± 0.7	30.0 ± 0.0	29.6 ± 0.8
30–34	-	29.0 ± 1.4	29.1 ± 0.8	-	-
35–39	-	28.8 ± 0.4	29.4 ± 0.8	29.7 ± 0.6	29.3 ± 1.2
40–44	-	27.0 ± 3.6	29.3 ± 0.8	29.0 ± 0.0	29.3 ± 0.6
45–49	30.0 ± 0.0	28.9 ± 1.1	29.1 ± 2.3	29.0 ± 1.4	30.0 ± 0.0
50–54	28.3 ± 1.0	28.7 ± 1.2	29.2 ± 0.6	-	29.3 ± 1.2
55–59	29.0 ± 0.0	28.8 ± 1.7	29.2 ± 0.8	29.0 ± 0.8	30.0 ± 0.0
60–64	27.8 ± 1.8	28.4 ± 2.1	29.3 ± 1.0	30.0 ± 0.0	29.8 ± 0.4
65–69	28.5 ± 1.3	27.7 ± 2.6	28.3 ± 2.8	28.0 ± 0.0	30.0 ± 0.0
70–74	26.5 ± 2.4	28.2 ± 2.1	28.3 ± 1.5	-	28.1 ± 2.7
75–79	26.8 ± 3.0	29.0 ± 0.8	28.5 ± 1.4	29.0 ± 0.0	29.0 ± 0.9
80–84	26.5 ± 1.9	25.5 ± 2.3	28.6 ± 1.4	-	27.8 ± 2.2
85–89	27.7 ± 0.6	25.7 ± 2.5	-	-	-
90–94	-	-	26.3 ± 2.9	-	-

The logarithm of 101 years of age and the square root of years of schooling were found to provide the best transformations of age and education. These transformed variables, sex, and their interaction terms were included in a simultaneous multiple linear regression analysis. Age, sex and education were all significant components of the final model that reported a value of *R*^2^ = 0.26 (Table 5).

**Table 5:** Results of multiple linear regression

Independent variables	Coefficients			*t*	*p*
	Unstandardised		Standardized		
	Beta	Standard error	Beta		
Constant	22.309	0.582		38.314	0.000
Ln 101-age	1.063	0.131	0.378	8.097	0.000
√Education	0.534	0.103	0.243	5.200	0.000
Sex	0.493	0.126	0.179	3.909	0.000

*R*^*2*^* 0.26*

Adjustment values are provided for all age *x* education ranges, for men and women separately, in Table 6 or may be calculated using the regression equation also displayed in Table 6. Adjusted scores lower than the outer tolerance limit of 26 are to be considered abnormal. Table 6 also reports equivalent scores.

**Table 6:**
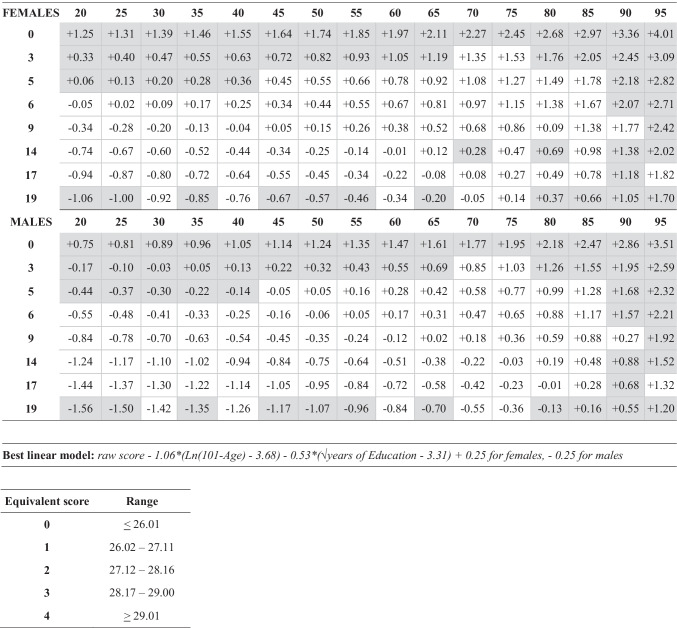
Correction grid, regression equation and equivalent scores for MMSE. Coefficients in grey cells were extrapolated by regression since no individual from those socio-demographic slots was enrolled in the study

## Discussion

Here, we report novel norms for Italian MMSE computed via multiple regression analysis in a sample of 361 young and old neurologically healthy and cognitively unimpaired individuals from Northern Italy and Italian speaking Southern Switzerland. Like in all previous Italian normative studies, age and schooling were significant predictors of performance on the test, with lower scores for older and less educated individuals. In addition, and unlike all prior studies, we also found a significant effect of sex: regression analysis indicated that women must be credited 0.5 points more than men (at all ages and educational levels).

The assumption that prompted an update of norms for Italian MMSE was a change in performance on the test in the general population over the years, putatively related to the educational and cultural progress occurred in the Italian society in the last three decades. In addition, exclusion of individuals with MCI, a concept introduced after publication of prior norms, may also have affected general performance of the new normative sample. Direct comparison of old and current findings is not straightforward due to differences in size, composition and stratification of study populations and to the use of slightly different versions of the test. Nevertheless, a contrast is possible with some age and education intervals from Measso et al.’s study (1993) and does confirm a trend towards an improvement in performance in our study sample, especially for older age groups (≥ 45 years) with a lower educational level (≤ 5 years), which showed up to a three-point increase in average raw scores in comparison with Measso’s participants. The observed increase in the cut-off point, from ≥ 22 [19] to ≥ 24 [20] to ≥ 26, clearly reflects such an increase. The recent norms by Carpinelli Mazzi et al. (2020a, b also had already identified a higher cutoff (≥ 25) than older studies.

The main limitations of the present study are the relatively small sample size. As explained in the “Introduction”, we focused our efforts on ruling out subjects with even mild impairment of cognitive functions, a key factor for ensuring high diagnostic accuracy of the new norms. To this aim, we assessed formally the main neuropsychological domains in all study entrants and applied a strict criterion for inclusion (all test scores had to be in the normality range). This procedure actually led to the identification of cognitive deficits in 10% of study candidates but limited the possibilities of high-volume recruiting. A second flaw of our study is the fact that some socio-demographic groups were poorly or not at all represented in our sample, namely very old individuals and poorly educated younger individuals. Subjects with the latter features are decreasing progressively in modern societies; hence, the need for norms for this socio-demographic group is going to be less and less stringent. Conversely, MMSE is used predominantly in elderly patients, and life expectancy of the general population is increasing constantly; therefore, the low prevalence of oldest-old in our study sample is more problematic. We exploited the data collected during our study to estimate adjustment values also for subjects above the age of 85, applying a robust statistical design that should ensure reliability, but clinicians and researchers willing to use our norms must be aware that, for older patients, such norms have been derived by extrapolation. A final, potential caveat of our work is the fact that data were collected in a restricted geographical area, along the borders between Northern Italy and Southern Switzerland. However, it is unsure whether or not the area of origin may have an impact on performance at simple tasks like those included in MMSE. As a matter of fact, Magni et al. [19] also collected their data in a small area between Brescia and Sondrio, yet their norms have been and are being used proficiently all over Italy. Recent norms for Southern Italy are anyway also available, at least for individuals between 50 and 79 years of age, since no subject above the age of 79 was included in the Southern study sample [4, 5].

## Conclusion

This paper reports on updated Italian norms for MMSE, providing novel adjustment coefficients (age, education and sex all had a significant effect on MMSE score) and cut-off value (≥ 26 /30) to be used for the screening and monitoring of global cognition in young and old patients of both sexes with various educational levels. Participants were carefully screened for mild cognitive deficits so that the normative sample was representative of the general cognitively healthy population, and the test protocol was partially amended (using the word ‘fiore’ instead of ‘pane’ in verbal recalls to reduce the common interference error ‘casa, *cane*, gatto’). Future studies should verify empirically the diagnostic validity of the new norms and of this slightly modified version of MMSE in clinical populations.

## Appendix

Appendix 1 MMSE administered in the normative study. See “Subjects and methods” section for administration and scoring instructions

## Footnotes

1 A fourth study was a re-analysis of Measso et al.’s data that calculated the fifth percentile norms as a step functions of age, rather than the more commonly used adjustment and cut-off values based on a linear regression model [13].
