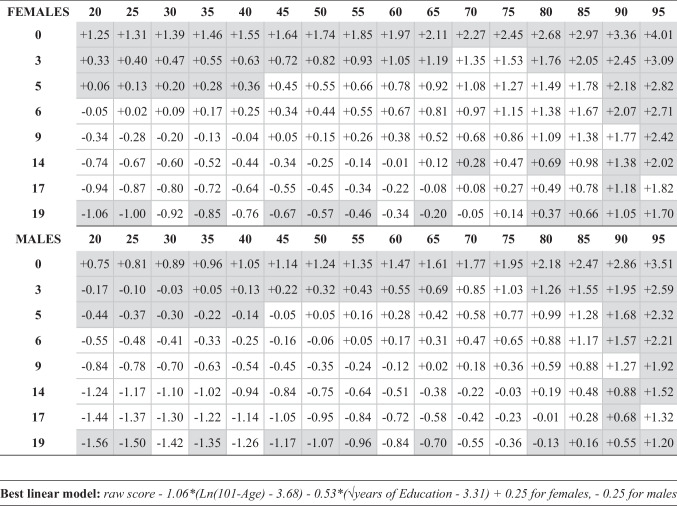# Correction to: Brand new norms for a good old test: Northern Italy normative study of MiniMental State Examination

**DOI:** 10.1007/s10072-024-07585-7

**Published:** 2024-05-17

**Authors:** Giuseppe Foderaro, Valeria Isella, Andrea Mazzone, Elena Biglia, Marco Di Gangi, Fabrizio Pasotti, Flavia Sansotera, Monica Grobberio, Vanessa Raimondi, Cristina Mapelli, Francesca Ferri, Valentina Impagnatiello, Carlo Ferrarese, Ildebrando Marco Appollonio

**Affiliations:** 1 EOC Ente Ospedaliero Cantonale, Lugano Regional Hospital, Lugano, Canton Ticino Switzerland; 2 Neurology Department, San Gerardo Hospital, Monza, Italy; 3 School of Medicine, University of Milano-Bicocca, Milan, Italy; 4 Milan Center for Neurosciences (NeuroMI), Milan, Italy; 5 Redaelli Geriatric Institute, Milan, Italy; 6 Neurocenter of Southern Switzerland, Lugano Regional Hospital, Lugano, Canton Ticino, Switzerland; 7 Psychology, Psychotherapy and Neuropsychology Center, Canton Ticino, Minusio Switzerland; 8 Department of Brain and Behavioural Sciences, University of Pavia, Pavia, Italy; 9 Clinical Neuropsychology Lab, Neurology Department and Clinical Psychology Unit, ASST Lariana, Como, Italy; 10 Psychology Service and Neurology Unit, ASST Crema, Crema, Italy

**Correction to** **:** **Neurological Sciences (2022) 43:3053–3063**

10.1007/s10072-021-05845-4

The original version of the above article contained a mistake in two cells of Table 6:

‘FEMALES’ table section: the adjustment value for 9 years of education and 80 years of age should be +1.09 instead of +0.09

‘MALES’ table section: the adjustment value for 9 years of education and 90 years of age should be +1.27 instead of +0.27.

Here is the updated table:

**Table:**